# Supplementary material for: Burden and determinants of scabies in Ethiopian school age children: A systematic review and meta-analysis with public health implications
Source: PLoS One. 2024 Dec 19;19(12):e0314882. doi: 10.1371/journal.pone.0314882 (PMC11658575; doi:10.1371/journal.pone.0314882)
Supplement: S3 File — (DOCX) [file pone.0314882.s003.docx]

**Annex II: NOS quality assessment score of cross-sectional studies**

| **Authors** | **Selection** | | | | **Comparability** | **Outcome** | | **NOS quality** | **Design** |
| --- | --- | --- | --- | --- | --- | --- | --- | --- | --- |
|  | **1** | **2** | **3** | **4** | **1** | **1** | **2** |  |  |
| **Tefera Haile et.al(1)** | A* | A* | A* | B* | A** | C* | B(null) | Good | Cross-sectional |
| **Abayneh Tunje et.al(2)** | A* | B(null) | A* | B* | A** | C* | A* | Good | Cross-sectional |
| **Desta Marmara et.al(3)** | A* | B(null) | A* | C* | A** | C* | A* | Good | Cross-sectional |
| **Yahya Kemer et.al(4)** | A* | A* | A* | B* | A** | C* | A* | Good | Cross-sectional |
| **Bisrat Misganaw et.al(5)** | A* | A* | A* | B* | A** | C* | A* | Good | Cross-sectional |
| **Sindayo Tefera et.al(6)** | A* | A* | A* | B* | A** | C* | A* | Good | Cross-sectional |
| **Stephen L. Walker et.al(7)** | C(null) | B(null) | A* | C(null) | B(null) | A** | B(null) | Unsatisfactory | Cross-sectional |
| **Gemechu Ararsa et.al(8)** | A* | A* | A* | B* | A** | C* | A* | Good | Cross-sectional |
| **Henok Dagne et.al(9)** | A* | A* | A* | B* | A** | C* | A* | Good | Cross-sectional |
| **Tarkie Abebe et.al(10)** | A* | A* | A* | B* | A** | C* | A* | Good | Cross-sectional |
| **Hiwot Hailu Amare et.al(11)** | A* | A* | A* | B* | A** | C* | A* | Good | Cross-sectional |
| **Yohannes Lulu et.al(12)** | A* | B(null) | A* | A** | A** | A** | A* | Very good | Cross-sectional |

**For cross-sectional studies**

- **Selection (maximum 5 stars)**

1=truly representative of the target population

2=Sample Size

3= Non-respondents

4= Ascertainment of the exposure (risk factor)

- **Comparability** **(Maximum 2 stars)**

1= results adjusted for relevant predicatores/risk factores/confounders.

- **Outcomes**  **(Maximum 3 stars)**

1=Assessment of outcomes ( Maximum 2 stars)

2= Statistical test (maximum 1 star)

**Newcastle-Ottawa scale adapted for Cross-sectional studies**

1. Very good studies 9-10 points of star.
2. Good studies 7-8 points of star.
3. Satisfactory studies 5-6 points of star.
4. Unsatisifactory studies 0-4 points of star.

**Annex III: NOS quality assessment score of case control studies**

| **Authors** | **Selection** | | | | **Comparability** | **Exposure** | | | **Scores (0-9)** | **Design** |
| --- | --- | --- | --- | --- | --- | --- | --- | --- | --- | --- |
|  | **1** | **2** | **3** | **4** | **1** | **1** | **2** | **3** |  | Case control |
| **Melat Wodaje et.al(13)** | A* | A* | A* | A* | A** | B* | A* | A* | 9 | Case control |
| **Kefele Ejigu et.al(14)** | A* | A* | A* | A* | A** | B* | A* | B(null) | 8 | Case control |
| **Yassin Zeyneba et.al(15)** | A* | B(null) | A* | A* | A** | B* | A* | A* | 8 | Case control |
| **Eden Gebre(16)** | A* | A* | A* | A* | A* | B* | A* | B(null) | 7 | Case control |

Note: A study can be given a maximum of one star for each numbered item within the Selection and Exposure categories. A maximum of two stars can be given for Comparability.

**Reference(citation)**

1. Haile T, Sisay T, Jemere T. Scabies and its associated factors among under 15 years children in Wadila district, Northern Ethiopia, 2019. Pan African Medical Journal. 2020;37(1).

2. Girma E, Churko C, Alagaw A, Haftu D, Tunje A, Tsegaye B. Prevalence of Scabies and Its Associated Factors Among School-Age Children in Arba Minch Zuria District, Southern Ethiopia, 2018. 2020.

3. Marmara D. Prevalence and Associated Factors of Scabies Among 5 to 14 Years Aged Children, in Boricha District, South Ethiopia: HUCMHS; 2019.

4. Hassen YK, Ame MM, Mummed BA, Yuya HM, Najib K, Abdella A, et al. Magnitude and Factors Associated With Scabies Among Primary School Children in Goro Gutu Woreda, East Hararghe Zone, Ethiopia. Int J Med Parasitol Epidemiol Sci Volume. 2022;3(4):84.

5. Misganaw B, Nigatu SG, Gebrie GN, Kibret AA. Prevalence and determinants of scabies among school-age children in Central Armachiho district, Northwest, Ethiopia. Plos one. 2022;17(6):e0269918.

6. Tefera S, Teferi M, Ayalew A, Belete T, Hadush H. Prevalence of scabies and associated factors among primary school children in Raya Alamata District, Tigray, Ethiopia, 2017/2018. J Infect Dis Epidemiol. 2020;6:154.

7. Walker SL, Lebas E, De Sario V, Deyasso Z, Doni SN, Marks M, et al. The prevalence and association with health-related quality of life of tungiasis and scabies in schoolchildren in southern Ethiopia. PLoS neglected tropical diseases. 2017;11(8):e0005808.

8. Ararsa G, Merdassa E, Shibiru T, Etafa W. Prevalence of scabies and associated factors among children aged 5–14 years in Meta Robi District, Ethiopia. Plos one. 2023;18(1):e0277912.

9. Dagne H, Dessie A, Destaw B, Yallew WW, Gizaw Z. Prevalence and associated factors of scabies among schoolchildren in Dabat district, northwest Ethiopia, 2018. Environmental health and preventive medicine. 2019;24:1-8.

10. Ferede TAWaAJ. Walle TA, Ferede AJ (2023) Prevalence of scabies and associated factors among Governmental Elementary School Students in Gondar town Northwest Ethiopia. J Pract Prof Nurs 7: 046. HSOA Journal of Practical & Professional Nursing 2023.

11. Amare HH, Lindtjorn B. Risk factors for scabies, tungiasis, and tinea infections among schoolchildren in southern Ethiopia: A cross-sectional Bayesian multilevel model. PLoS neglected tropical diseases. 2021;15(10):e0009816.

12. Lulu Y, Tolesa G, Cris J. Prevalence and associated factors of skin diseases among primary school children in Illuababorzone, Oromia Regional State, South West Ethiopia. Indo Am J Pharm Res. 2017;7(1):7374-83.

13. Reta MW, Derseh BT, Sahilu BY. Determinants of scabies among primary school children in Habru district: A case-control study. 2020.

14. Ejigu K, Haji Y, Toma A, Tadesse BT. Factors associated with scabies outbreaks in primary schools in Ethiopia: a case–control study. Research and reports in tropical medicine. 2019:119-27.

15. Yassin ZJ, Dadi AF, Nega HY, Derseh BT, Asegidew W. Scabies Outbreak Investigation among “Yekolo Temaris” in Gondar Town, North Western Ethiopia, November 2015. Electronic Journal of Biology. 2017;13(3):203-9.

16. Gebre E. Determinants of scabies infection among school agechildren in Siraro district west Arsi zone, Oromia region, Ethiopia,2021: Unmatched case control study. 2021.
